# Detection of Road Crack Images Based on Multistage Feature Fusion and a Texture Awareness Method

**DOI:** 10.3390/s24113268

**Published:** 2024-05-21

**Authors:** Maozu Guo, Wenbo Tian, Yang Li, Dong Sui

**Affiliations:** 1 School of Electrical and Information Engineering, Beijing University of Civil Engineering and Architecture, Beijing 102616, China; guomaozu@bucea.edu.cn (M.G.); 2108550021029@stu.bucea.edu.cn (W.T.); suidong@bucea.edu.cn (D.S.); 2 Beijing Key Laboratory for Intelligent Processing Methods of Architectural Big Data, Beijing University of Civil Engineering and Architecture, Beijing 102616, China

**Keywords:** deep learning, transformer, crack detection, segmentation, attention mechanism

## Abstract

Structural health monitoring for roads is an important task that supports inspection of transportation infrastructure. This paper explores deep learning techniques for crack detection in road images and proposes an automatic pixel-level semantic road crack image segmentation method based on a Swin transformer. This method employs Swin-T as the backbone network to extract feature information from crack images at various levels and utilizes the texture unit to extract the texture and edge characteristic information of cracks. The refinement attention module (RAM) and panoramic feature module (PFM) then merge these diverse features, ultimately refining the segmentation results. This method is called FetNet. We collect four public real-world datasets and conduct extensive experiments, comparing FetNet with various deep-learning methods. FetNet achieves the highest precision of 90.4%, a recall of 85.3%, an F1 score of 87.9%, and a mean intersection over union of 78.6% on the Crack500 dataset. The experimental results show that the FetNet approach surpasses other advanced models in terms of crack segmentation accuracy and exhibits excellent generalizability for use in complex scenes.

## 1. Introduction

The long-term use of roads is subject to various influencing factors, resulting in structural damage, including load, temperature, and seismic effects. Damage to roads negatively affects their functionality, which may threaten human safety in severe cases. Thus, regular damage detection and maintenance tasks are important for improving road safety and durability [1]. Early road damage detection methods mainly relied on manual inspection to determine structural damage amounts, distributions, and sizes. The results depend entirely on the knowledge and experience of the inspectors, exhibiting strong subjectivity, high time consumption, and high labor intensity. This approach is susceptible to factors such as visual fatigue and working at heights, which can lead to omissions or misjudgments [2] that make it difficult to satisfy the extensive detection needs of practical engineering scenarios. In addition to manual road crack detection methods, crack segmentation methods rely on available conventional image algorithms, including edge detection and image processing strategies [3,4].

Regrettably, while these methods have surpassed manual crack detection in terms of accuracy, most fail to address the challenge posed by complex noise, which may result in limitations such as low accuracy and efficiency. In addition, these algorithms have several shortcomings: they lack reliable feature representation capabilities and neglect the interdependence between cracks. The above-mentioned methods are usually based on shallow features and image processing techniques; however, these methods are limited in their ability to address scenes with complex crack images, and they cannot satisfy the feature expression and generalization requirements imposed by complex crack image scenes.

**Figure 1:**
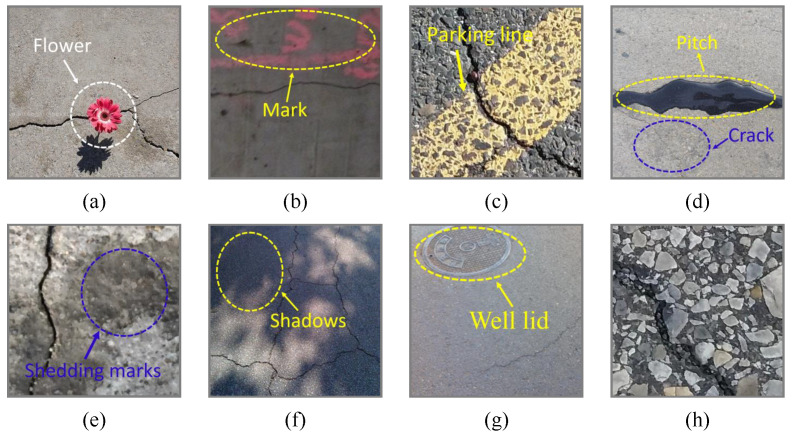
Road crack images contaminated with various complex noises: (**a**) a flower, (**b**) a mark, (**c**) a parking line, (**d**) pitch, (**e**) shedding marks, (**f**) shadows, (**g**) a well lid, and (**h**) a complex background.

In recent years, deep learning image segmentation has emerged as the mainstream method. In segmentation scenarios, cracks typically exhibit unique textural and shape characteristics. Variations in the size, shape, and orientation parameters result in distinct texture features. Additionally, the low separability between different noise categories and cracks in crack images increases the difficulty of performing semantic segmentation. For example, cracks may have similar colors and shapes as the surrounding road surface, rocks, or other ground objects, making them visually challenging to distinguish. Moreover, images of high resolution contain a wealth of intricate details, leading to various scale issues in semantic segmentation tasks. These images may exhibit different features at various scales, often confusing multiscale segmentation models. Road surfaces are mainly divided into concrete and asphalt surfaces, which display smoothness and porosity differences. Asphalt surfaces are rougher and have more complex backgrounds than concrete surfaces. As shown in Figure 1, road crack images feature uneven illumination conditions, complex topological structures, low contrast levels, diverse textures, and noise interference. These characteristics pose great challenges to deep learning algorithms [5].

The keys to successfully segmenting crack images lie in obtaining spatial information for establishing long-range dependencies and establishing a global relationship based on the observed crack regions [6]. Spatial information encompasses the textures and shapes of road cracks. Networks with strong feature learning capabilities are essential for obtaining comprehensive spatial information concerning cracks. Nevertheless, traditional convolutional neural network (CNN) architectures and their variants are limited in terms of acquiring spatial information, as convolution and pooling operations reduce image resolutions, resulting in the diminishment of spatial detail. Considering the typically long and narrow shapes of cracks, it is difficult to accurately delineate their boundaries by relying solely on local contextual information. Thus, to effectively comprehend the connection between the individual crack and the class attribute of the overall crack region, it is essential to become more globally aware of cracks. CNNs and their adaptations primarily enhance long-range dependencies by modifying convolutional operations [7] and by incorporating attention mechanisms [8]. Nonetheless, attention mechanisms are limited to CNNs based on a priori knowledge and do not consider entire feature sets. In contrast, the transformer architecture processes images in a serialized manner and uses positional embeddings to describe spatial relationships, acquiring more comprehensive spatial information without convolution or pooling operations. Moreover, this approach exhibits significant advantages in enhancing long-range dependencies and global awareness due to the use of multihead attention mechanisms.

In summary, this paper proposes a method for the detection and segmentation of road cracks based on a Swin transformer [9]. The approach adopts a multipath fusion network architecture, with a tiny Swin-T serving as its backbone network. It also uses a texture unit [10] with stacked convolution layers to extract crack texture information. Furthermore, a refinement attention module (RAM) is developed to merge features output by Swin-T from each stage, and a panoramic feature module (PFM) is developed for fusing the features output by Swin-T and the texture unit. The overfitting residuals are reduced using a cascade pyramid architecture (CPA). Finally, the segmentation results are optimized by a fully connected conditional random field (FC-CRF).

Our contributions are as follows:

(1) We develop an automatic road crack image detection and segmentation method based on Swin-T. It leverages Swin-T and a texture unit to capture multilevel features and texture features, respectively, from input images. This architecture utilizes convolution operations at various levels to acquire multilevel semantic perceptual information.

(2) We integrate the concept of a multipath network to fuse the features output from different levels. This fusion process combines multilevel and texture features by introducing the RAM and the PFM we designed. This approach not only enhances the generalizability of the network but also reduces overfitting residuals.

(3) We use an FC-CRF to optimize the segmentation results. This optimization process efficiently eliminates misclassification and refines the edges of the segmentation results, thus enhancing both their completeness and accuracy.

## 2. Related Work

Currently, the available deep learning road crack detection models can be broadly categorized into two types: those based on CNN architectures and those based on transformer architectures. CNN-based crack image segmentation methods have surpassed the traditional segmentation approaches in terms of performance [11]; however, they still face challenges when attempting to catch long-range dependencies and fine-grained crack details. Conversely, the introduction of transformer architecture has provided a new outlook for crack image segmentation tasks, yielding enhanced accuracy and efficiency by enabling direct prediction and global context reasoning.

### 2.1. CNN-Based Crack Segmentation Models

Researchers have attempted to enhance the segmentation efficacy in crack images for CNN-based approaches by utilizing different network structures. Wang et al. applied CNNs to detect pavement crack tasks and employed principal component analysis (PCA) for classifying pavement cracks [12]. Kim et al. introduced a CNN-based architecture for segmenting surface cracks in concrete. This model enables civil structures to be easily monitored using low-power computing devices [13]. Nguyen et al. developed a CNN model for crack segmentation. This CNN architecture can detect all image patches containing cracks. Nonetheless, one limitation of this model lies in its inability to localize cracks with precision, as observed in the ground truth [14]. Yang et al. used fully convolutional networks (FCNs) for the semantic identification and segmentation of cracks across various scales and integrated morphological operations for deriving geometric features, including length and width information, straight from visual data, bypassing manual measurements [15]. Reference [16] implemented an improved FCN by fine-tuning a smaller training model based on DenseNet-121 to conduct pixel-level segmentation on various damaged surfaces. Reference [17] indicated that crack segmentation performance can be enhanced by adding deeper backbone networks to an FCN model, as well as by adding skip connections to U-Net.

Despite the semantic crack image segmentation performance improvements achieved by these deep learning models, to some extent, they still face challenges. For example, traditional CNN and FCN methods often fail to effectively catch long-range dependencies and fine-grained crack details, limiting the accuracy of the segmentation process. In addition, these methods may not fully utilize the feature information located at different scales of multiscale crack images.

### 2.2. Transformer-Based Crack Segmentation Models

A transformer is a neural network architecture that was initially proposed for natural language processing (NLP) tasks, and it has transcended its domains to find extensive applications in image processing, video analysis, and more. This network model encompasses a series of multiple encoder and decoder layers, with each layer comprising self-attention modules and feedforward neural network layers. The encoder layers process the input sequence, thereby generating a set of feature vectors. These feature vectors serve as the basis upon which decoder layers construct the output sequence. Carion et al. [18] developed a simple and flexible DEtection TRansformer (DETR) architecture for object detection tasks based on transformers and a bipartite matching loss. This approach eliminates the need for handcrafted processes and achieves competitive performance on challenging datasets. Dosovitskiy et al. [19] proposed the Vision Transformer (ViT) model marked transformer architecture, can be applied to computer vision tasks. Xie et al. [20] introduced SegFormer, a method that exhibits outstanding performance in high-speed image segmentation scenarios. SegFormer is a simple and efficient semantic segmentation framework that integrates transformers alongside minimalist MLP decoders. Despite the ability of a range of one-dimensional input transformers to simulate the global context in all stages, the ability of SegFormer to capture local details is limited. Reference [21] developed a semantic segmentation model called SETR, which replaces the traditional stacked convolution layers with a pure transformer encoder. SETR has achieved considerable performance on multiple benchmarks. Wang [22] designed an artificial neural network based on a transformer specifically for recognizing cracks in concrete, using a hierarchical transformer architecture. This design employs transformer encoders to generate multiscale features and adopts a top-down approach to progressively up-sample and merge the features derived from the deepest encoder layer.

## 3. FetNet

To address the above issues and capture more detailed features from input images while establishing long-range global relationships, we propose the FetNet architecture. We employ Swin-T as the backbone network to acquire multilevel crack semantic perceptual information. We propose using RAM and FPM to establish long-range global relationships, integrate a multipath network concept to fuse the output of the features at various levels, and enhance the texture and edge features of cracks.

### 3.1. Network Architecture

**Figure 2:**
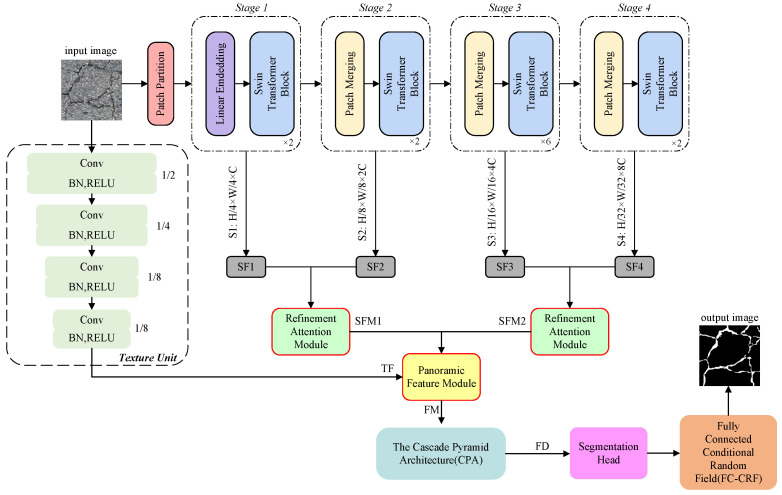
The architecture of FetNet.

The comprehensive architecture of FetNet is depicted in Figure 2. Firstly, Swin-T is employed to execute multistage feature transformations, computing self-attention to extract semantic features, wherein the receptive field of each patch expands while the feature dimension decreases. This process generates four feature representations, namely SF1, SF2, SF3, and SF4. Second, a RAM is proposed to pair and merge different semantic features extracted by Swin-T in four stages, since different resolution features possess responsiveness to different crack feature categories. Enhancing global dependence of semantic features and attention mechanisms results in feature maps SFM1 and SFM2. Third, a texture unit processes the input image to extract texture features, resulting in feature TF. Subsequently, the PFM is proposed to concatenate the up-sampled SFM1, SFM2, and TF, producing feature map FM that contains long-range dependency and texture information. Fourth, we process FM through CPA to enhance the network’s capacity for building global relationships and differentiate between multiscale categories. This process ultimately yields fusion map FD that possesses all the features, and the fused features are processed by a segmentation head to yield segmentation maps. Finally, segmentation results optimized by an FC-CRF are produced.

### 3.2. Swin Transformer-Based Feature Extraction Block

**Figure 3:**
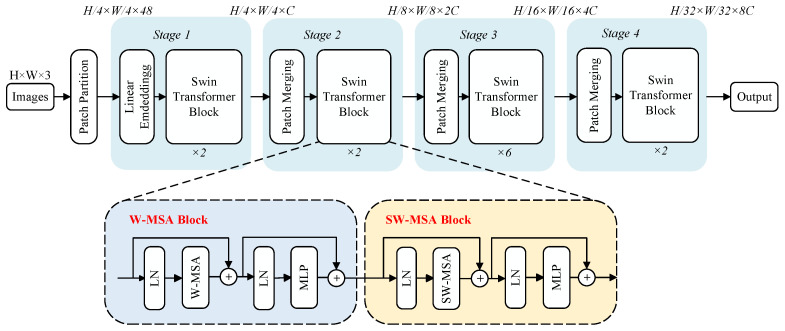
The architectures of Swin-T and two successive Swin-T blocks.

Transformer networks face challenges in image processing due to limitations in handling varied visual elements and high-resolution complexity, leading to inefficient performance and high computational burden when using the global self-attention mechanism of a transformer, especially in scenarios involving segmentation and detection. The Swin transformer addresses these issues by introducing layered feature extraction and sliding window operations, as illustrated in Figure 3. Layered extraction reduces the feature map size and expands receptive fields as network depth increases, enabling hierarchical feature extraction. The sliding window operation mitigates the issue in the ViT model, where the correlations between local windows are ignored. By confining the attention calculations within non-overlapping local windows and allowing the connection between these windows, the computational complexity remains constant. Additionally, irrelevant parts are masked out by assigning them attention weights of 0. This approach empowers the Swin transformer to effectively address missions that involve segmentation and detection.

**Figure 4:**
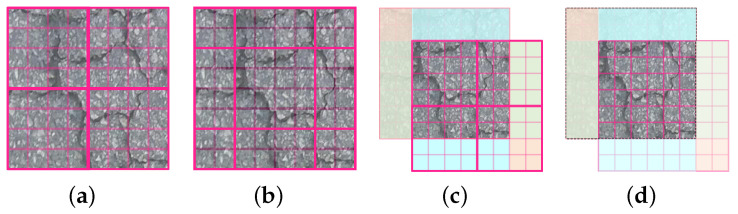
The shifted windows of multihead self-attention.

The Swin-T architecture comprises one patch partitioning step followed by four stages, as illustrated in the upper part of Figure 3. The patch partitioning operation segments the input RGB images into non-overlapping patches. Stage 1 includes linear embedding and two Swin-T blocks. The linear embedding process embeds the patches into vectors. The structure of a Swin-T block is illustrated in the lower part of Figure 3. These blocks apply window-based multihead self-attention (W-MSA and SW-MSA) to calculate pixel similarities within windows. The latter uses shifted windows to capture a broader context. After initial layer normalization and attention calculations, residual connections, layer normalization, and MLP with GELU activation combine and refine features, and then transform the matrix-derived parameters into their respective probability outputs. Finally, residual calculations are applied again. The subsequent stages include a patch merging module and Swin-T blocks. The patch merging module down-sample inputs, adjusts channel counts, and creates a hierarchical structure, conserving computation. The sliding window process exemplified in Figure 4 entails partitioning the image (Figure 4a); shifting the window (Figure 4b), where the shifted portions are marked in blue, pink, and green; reconstructing shifted images into new windows (Figure 4c); performing self-attention on them; and finally restoring the shifted portions to their original positions (Figure 4d).

### 3.3. Texture Unit

The texture unit harnesses a convolutional network architecture to extract textural and edge information from crack images. This pathway constructs a sequence of four distinct convolutional layers, collectively denoted as T, to meticulously capture the intricate textural patterns. Each stage T incorporates a composite function that includes a convolutional layer, batch normalization (BN), and ReLU activation for effective feature extraction [10]. The texture unit is as follows:

(1) $$TF\left(X\right)=T_{4}\left(T_{3}\left(T_{2}\left(T_{1}X\right)\right)\right).$$

Starting with $T_{1}$, a convolutional layer with a kernel size of 7 and a stride of 2 is employed to transition from an initial channel dimension of 3 to 64, thus widening the receptive field and enhancing the representation of fine-grained textures. Moving onto $T_{2}$ and $T_{3}$, the kernel size is reduced to 3 with a stride of 2, maintaining a consistent channel dimension of 64, which further refines the captured texture details while preserving structural information around the edges. Lastly, $T_{4}$ concludes the textural pathway with a standard 1×1 convolution, having a stride of 1, doubling the channel dimension from 64 to 128. Throughout this process, the texture path progressively down-scales the input image by a factor of 8 while simultaneously enriching the feature representation with a channel count of 128. Consequently, the outputted textural and edge feature map encapsulates a highly condensed yet informative representation of the crack image’s salient attributes.

### 3.4. The Refinement Attention Module

**Figure 5:**
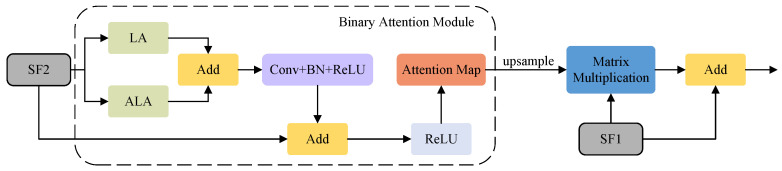
The refinement attention module and the binary attention module.

**Figure 6:**
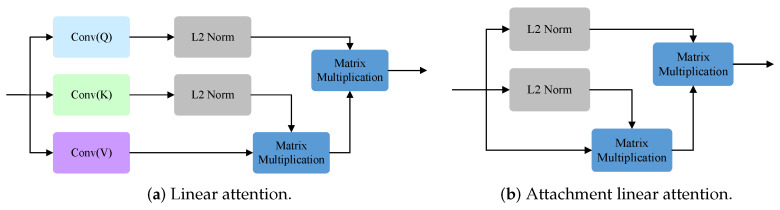
The linear attention and the attachment linear attention.

We designed the RAM to fuse different features, and its purpose within the entire architecture is to merge the features extracted from various phases of the Swin-T, thereby enhancing its crack segmentation performance. The RAM employs our proposed binary attention module (BAM) to bolster the spatial relationships of SF2. Swin Transformer significantly reduces memory overhead by applying multihead attention mechanisms within its limited window. However, this alternating rule and shift window execution inevitably limits the global modeling ability of the model. To better solve this limitation, we designed BAM, which integrates LA in the spatial dimension and introduces ALA in the channel dimension to consider the interrelationship between pixels, thus strengthening the global dependence of semantic features [23]. By taking advantage of this global dependence, BAM can gain a deeper understanding of the overall background information of pavement cracks. Global multiscale information is transmitted to LA and ALA for attention enhancement, while LA and ALA have long-term dependencies in the spatial and channel dimensions. Models with LA and ALA can both capture the details of various feature structures and also provide insight into the internal relationships between these features to achieve more accurate detection results. Next, a convolutional layer that incorporates BN and ReLU is deployed. Subsequently, an addition operation is performed between the processed SF2 and the original SF2 to obtain the attention SF2. Following this, a ReLU activation function is employed to yield the attention map. Thereafter, a matrix multiplication operation is performed between the up-sampled attention map of SF2 and SF1, resulting in the attentional version of SF1. Finally, an addition operation is applied to merge the original SF1 with the attentional version of SF1 to obtain SFM1. The same operation leads to SFM2. RAM and BAM are illustrated in Figure 5, while LA and ALA are illustrated in Figure 6. The mathematical representation of the RAM is as follows:

(2) RAM(SF1,SF2)=SF1+SF1·U(BAM(SF2)),

where U signifies the up-sampling operation, and the scale factor is set to 2.

The BAM is described as follows:

(3) BAM(X)=ReLU(X+Conv(BN(ReLU(ALA(X)+LA(X))))),

where Conv denotes a standard convolution with a stride of 1.

### 3.5. The Panoramic Feature Module

**Figure 7:**
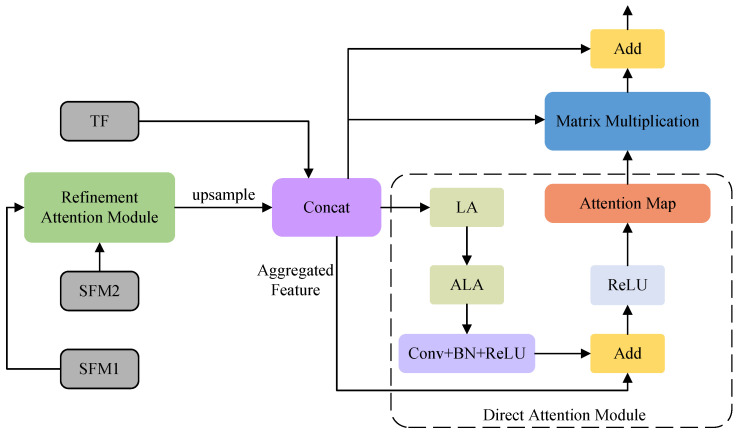
The panoramic feature module and the direct attention module.

Throughout the network, the PFM utilizes the advantages offered by both the features extracted from Swin-T and the texture features to create a powerful feature representation, as depicted in Figure 7, where the input to PFM consists of SFM1, SFM2, and TF. To combine these features, the RAM is initially employed to merge SFM1 and SFM2. Subsequently, the fused feature undergoes up-sampling and is concatenated with TF, resulting in an aggregated feature (AF). Finally, the direct attention module (DAM) minimizes the residual error when fitting the AF. The representation of the PFM pipeline can be described as follows:

(4) PFM(AF)=AF(DAM(AF)+AF)+AF

(5) AF(TF,SFM1,SFM2)=C(U(RAM(SFM1,SFM2)),TF),

where C denotes the concatenation operation and U signifies the up-sampling procedure with a scaling factor of 2.

The pipeline of the DAM can be described as follows:

(6) DAM(X)=ReLU(X+Conv(BN(ReLU(ALA(LA(X)))))).

The PFM involves the utilization of LA and ALA to amplify the spatial connections of the AF, thus inhibiting the fitting residual. Next, a convolutional layer that incorporates BN and ReLU is deployed. Thereafter, an additional operation is performed between the processed AF and the original AF to obtain the attention AF. Following this, a ReLU activation function is applied to yield the attention map. Subsequently, a matrix multiplication operation combines the aggregated features with the attention map, acquiring the attentional AF. Finally, the original AF and the attentional AF are fused to achieve FM through the addition operation.

### 3.6. The Cascade Pyramid Architecture

**Figure 8:**
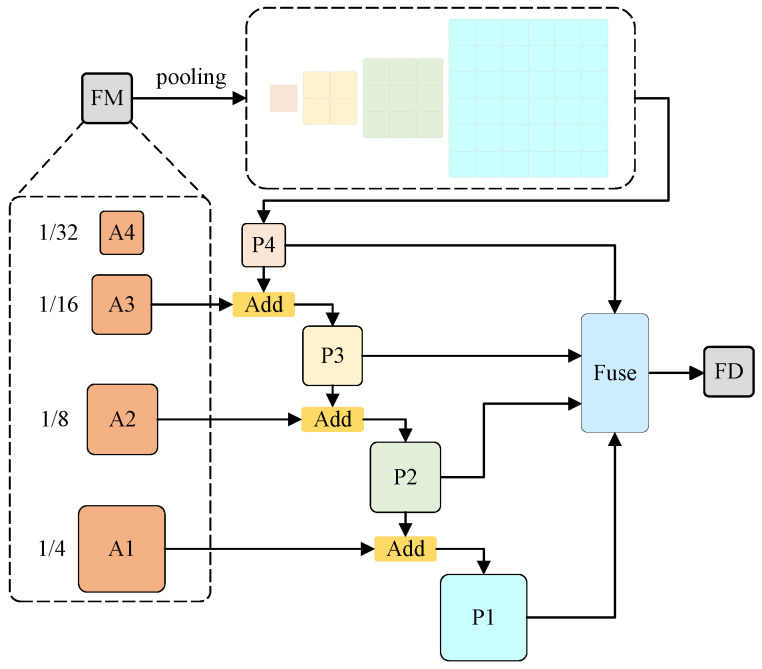
The cascade pyramid architecture.

In the entire network, the CPA initially endows the model with multiscale receptive fields to enhance its multiscale category recognition ability [24], then, through additive operations, to progressively integrate features from each stage, resulting in a fusion map that encompasses all features. The structural process of the CPA is visually represented in Figure 8. Feature map FM employs various pooling scales, including 1, 2, 3, and 6, to perform global pooling. This approach can aggregate the contextual information of different regions, accounting for both the global features and the locally particular information, thereby enhancing the ability of the model to distinguish between multiscale categories and resulting in improved segmentation performance. Then, additive operations to progressively integrate characteristics from each output stage. Specifically, input A3 (1/16) is combined with P4 (1/32) to produce a fusion feature P3 (1/16), and P2 (1/8) and P1 (1/4) are obtained in the same way. Subsequently, fusion blocks are used to fuse P1, P2, P3, and P4, yielding a fusion diagram FD that encompasses all features.

### 3.7. The Fully Connected Conditional Random Field

A conditional random field (CRF) [25] is a classic statistical model that generates directionless probabilistic maps based on the hidden Markov model and maximum entropy model, and it is widely applied in image segmentation tasks. A CRF is limited to its neighboring CRFs and requires significant computational power, making it difficult to describe relationships between pixels that are far apart. In the FC-CRF, each pixel within the graph model is considered a node, and each pair of nodes is interconnected by an edge. It is assumed that each node within the graph is connected with every other pixel in the input images. By utilizing higher-order potentials, this method not only considers the neighborhood information of pixels but also effectively exploits contextual information within the image, implementing long-range dependency reasoning, thereby improving segmentation accuracy [26]. This study employs the FC-CRF to solve the problems of fuzzy edges, large deviations, fragmentation, and the presence of small pseudo-regions in the segmentation outputs that can arise due to the lack of smoothness constraints. The computational procedure for the FC-CRF is delineated as follows. The energy function of the model is as follows:

(7) $$E\left(x\right)=\sum_{i}\psi_{u}\left(x_{i}\right)+\beta \sum_{i<j}\psi_{p}\left(x_{i},x_{j}\right).$$

Here, *x* signifies the label assigned to each pixel, where *i*, *j* are within [1, N]; $\psi_{u}\left(x_{i}\right)$ represents a pretrained classifier capable of computing the expense associated with assigning a particular label to a node, taking into account its feature characteristics; and $\psi_{p}\left(x_{i},x_{j}\right)$ is expressed as a summation of the Gaussian function with weights and can be described as follows:

(8) $$\psi_{p}\left(x_{i},x_{j}\right)=\delta \left(x_{i},x_{j}\right)k\left(f_{i},f_{j}\right)$$

(9) $$k\left(f_{i},f_{j}\right)=\sum_{m=1}^{k}w_{m}k_{m}\left(f_{i},f_{j}\right).$$

Here, δ signifies the second-order Potts function, which captures the interaction between neighboring pixels; *k* denotes the total count of kernels; $f_{i}$ and $f_{j}$ represent the feature vectors to the pixel nodes within the feature domain; $w_{m}$ represents the weight associated with the relevant Gaussian kernel, which plays a pivotal role in determining the degree of influence it exerts on the overall computation; and $k_{m}$ refers to the *m*-th Gaussian kernel function, each designed to measure the similarity between the feature vectors in a manner akin to a radial basis function. The Gaussian kernel accounts for both the bilateral positional and color relationships. It can be expressed as follows:

(10) $$\begin{array}{c} k_{m}\left(f_{i},f_{j}\right)=w_{1}\exp\left(-\frac{p_{i}-p_{j}^{2}}{2\sigma_{\alpha }^{2}}-\frac{l_{i}-l_{j}^{2}}{2\sigma_{\beta }^{2}}\right)+w_{2}\exp\left(-\frac{p_{i}-p_{j}^{2}}{2\sigma_{\gamma }^{2}}\right). \end{array}$$

In Equation (10), the appearance core is constructed based on the first core positional information *p* of two pixels, along with their color intensity *l*. This enables the easy classification of pixels with analogous spectral traits that are near the central pixel into the same category. The second core is referred to as the smoothing core, which solely accounts for the positions of pixels and results in smoother isolation regions. The variables $\sigma_{\alpha }$, $\sigma_{\beta }$, and $\sigma_{\gamma }$ serve as hyperparameters that regulate the scale of the Gaussian kernel. FC-CRF effectively leverages the interplay between pixels and fully connected pairwise potentials, capitalizing on the features extracted from the image, ultimately leading to the precise segmentation of crack images.

### 3.8. Loss Function

The proportion of crack pixels within a given crack image is smaller compared to non-crack pixels, potentially leading to a class imbalance in the total loss calculation. To address this issue, this study employs the focal loss (FL) function [27]. The FL is a modification of conventional cross-entropy loss, aiming to mitigate the influence of easily classifiable instances and to promote a greater emphasis on challenging samples in training. Its mathematical formulation can be represented as follows:

(11) $$FL\left(p_{t}\right)=-\alpha_{t}{\left(1-p_{t}\right)}^{\gamma }\log\left(p_{t}\right).$$

Here, the weight factor $\alpha_{t}$ is set to 0.25, and ${\left(1-p_{t}\right)}^{\gamma }$ denotes the modulation factor. By adding ${\left(1-p_{t}\right)}^{\gamma }$ to the model, a loss reduction is observed for samples with high prediction probabilities, while for samples with low prediction probabilities, the loss increases. Consequently, the model enhances the emphasis on learning from positive sample crack pixels. When y=1, $p_{t}=p$; when y=0, $p_{t}=1-p$. Here, *y* represents the actual class label within (0,1). Additionally, *p* lies within (0,1), which denotes the model’s estimated probability that the label is y=1. Also, t∈(0,1) denotes the indicator of category, with t=0 representing background pixels and t=1 denoting crack pixels. In this study, γ is set to 2.

## 4. Experiments

### 4.1. Datasets

To assess the effectiveness and generalizability of the FetNet model, we select four public road crack datasets for our experimental evaluations. The labels for all the publicly available datasets are sourced from their original images.

The publicly available DeepCrack dataset (544×384 pixels) [28] contains 537 high-quality crack images of various scenes with different scales, providing comprehensive representations of crack features. The dataset includes diverse surfaces and scenes, with crack images captured from surfaces such as concrete, brick, and stone. Furthermore, these images cover a wide range of capture scenarios, including those with different angles, lighting conditions, and environmental backgrounds. The crack images in DeepCrack encompass various types of cracks, including those with different shapes, sizes, and orientations.

The publicly available GAPs384 dataset (1920×1080 pixels) [29] exclusively contains 1969 asphalt crack images. Highly trained operators manually annotated the images at a high-resolution scale and delineated the genuine damaged areas using bounding boxes. The cracks in this dataset include single and multiple cracks, longitudinal and lateral cracks, alligator cracks, and sealed or filled cracks.

The 3368 crack images in the public Crack500 dataset (2000×1500 pixels) [30] are all asphalt cracks. Multiple annotators annotated each image. To optimize the trade-off between computational complexity and segmentation precision, each sample generates a 99×99 pixel image patch from three channels (RGB).

The publicly available CFD dataset [31] has images with sizes of 480×320 pixels and contains 118 crack images of asphalt and concrete that roughly reflect the urban road conditions in Beijing, China. Each image has manually annotated ground truth contour lines. The image widths range from 1 to 3 millimeters. These images include noise such as shadows, oil stains, and watermarks.

### 4.2. Implementation Details

#### 4.2.1. Computational Platform

The algorithms used in our study are programmed in Python 3 and imported into the PyTorch machine learning library. The operations are performed on an Ubuntu 20.04.5 LTS operating system with a 3.4 GHz Intel Xeon Gold i7-6135 CPU with 754 GB of RAM and a 32 GB NVIDIA Tesla V100S GPU.

#### 4.2.2. Parameter Settings

The weighted adaptive moment estimation (AdamW) optimizer is selected to reduce the computational loss and accelerate the convergence process, with a momentum of 0.9, a weight decay rate of 0.0001, and a batch size of 8. The base learning rate is set to 0.001, and the maximum number of training epochs is 300. The segmentation head is a critical component for utilizing the primary feature. After the backbone network extracts features, the consistency between the input and output images is ensured by the segmentation head, which conducts classification at the pixel level. We opt for the seghead as the segmentation head. In order to avoid overfitting, we employ data augmentation techniques, including image flipping, rotation, scaling, and translation, to expand the number of training samples. These augmented samples enhance the model’s ability to learn and understand image features more efficiently. Furthermore, we divide the dataset into training, testing, and validation sets, with ratios of 0.7, 0.2, and 0.1, respectively. At the same time, the focal loss function is used, and the early stopping strategy is adopted. The parameter quantity of FetNet is 59.83 M, and FLOPs is 70.6 G. We conducted a performance comparison of FetNet with that of the FCN, U-Net, DANet, ApcNet, CcNet, GcNet, HrNet, DnlNet, OcrNet, and Resnest crack segmentation algorithms.

### 4.3. Evaluation Criteria

The performance of each network is evaluated using precision (P), recall (R), *F*1 score (F1), accuracy (ACC), and mean intersection over union (mIoU) metrics, which are computed based on the accumulated confusion matrix as follows:

(12) $$precision=\frac{TP}{TP+FP}$$

(13) $$recall=\frac{TP}{TP+FN}$$

(14) $$F1=\frac{2\times precision\times recall}{precision+recall}$$

(15) $$accuracy=\frac{TP+TN}{TP+FN+TN+FP}$$

(16) $$\begin{array}{c} mIoU=\frac{1}{2}\frac{TP}{TP+FN+FP}, \end{array}$$

where TP, FP, FN, and TN indicate the numbers of true positive, false positive, false negative and true negative results, respectively.

### 4.4. Results and Discussion

**Figure 9:**
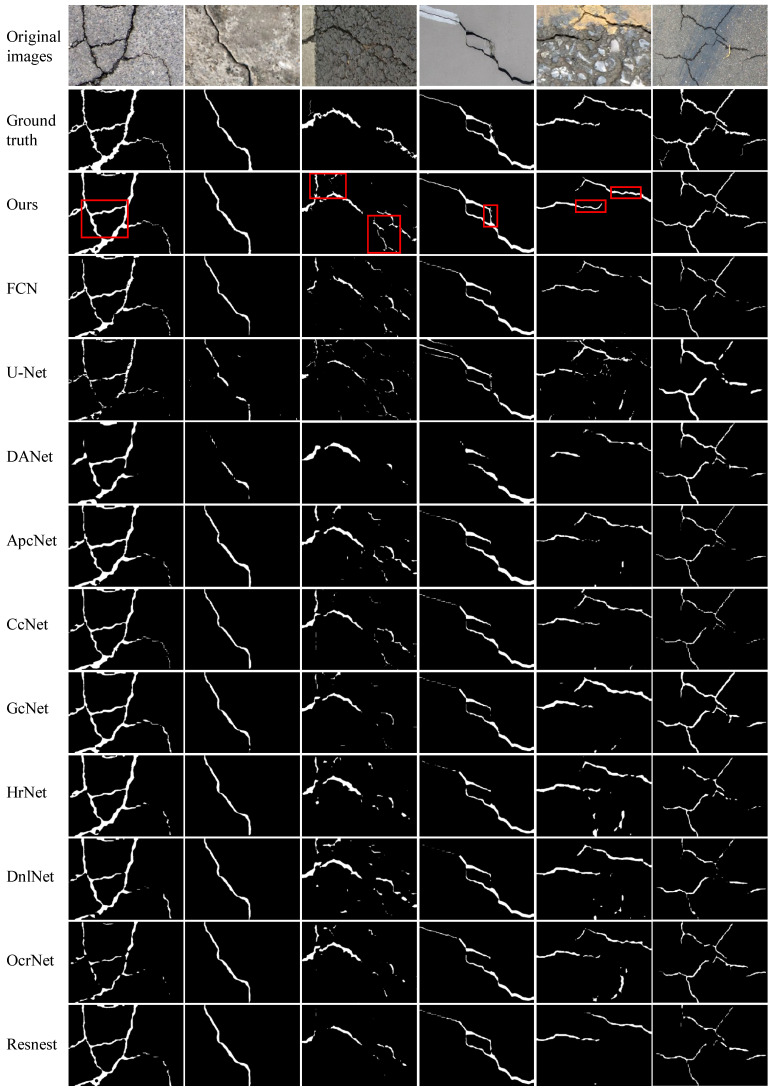
The prediction results obtained by the compared methods used on the DeepCrack dataset.

**Figure 10:**
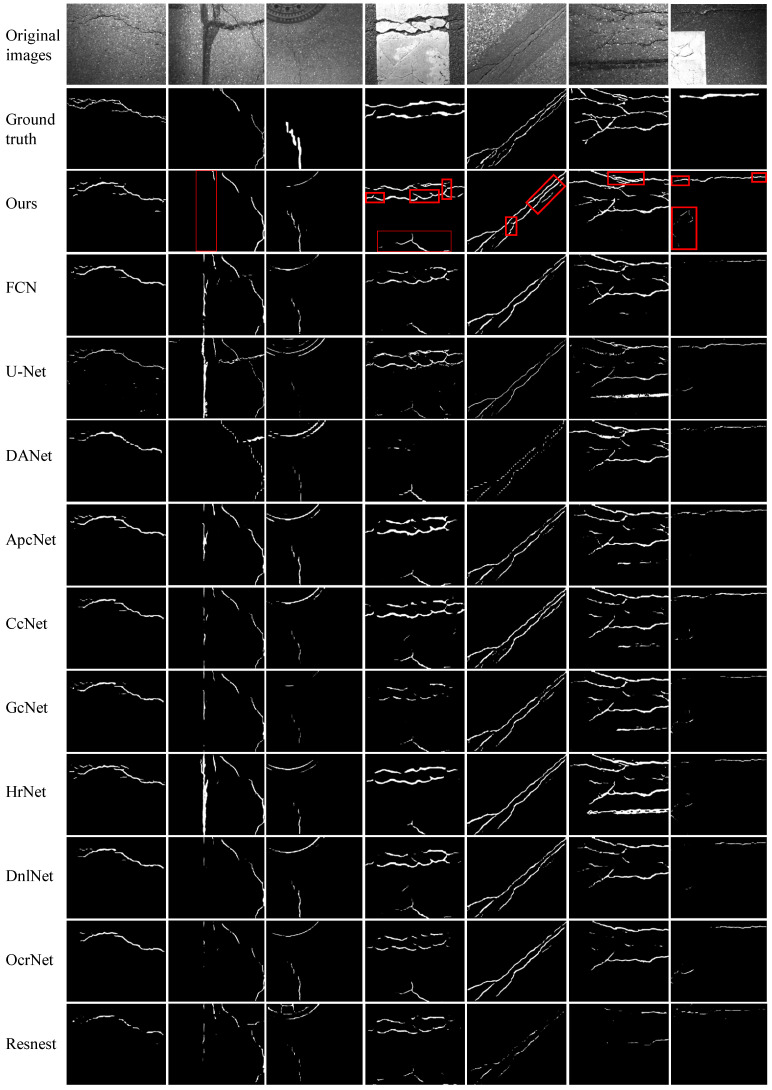
The prediction results obtained by the compared methods used on the GAPs384 dataset.

**Figure 11:**
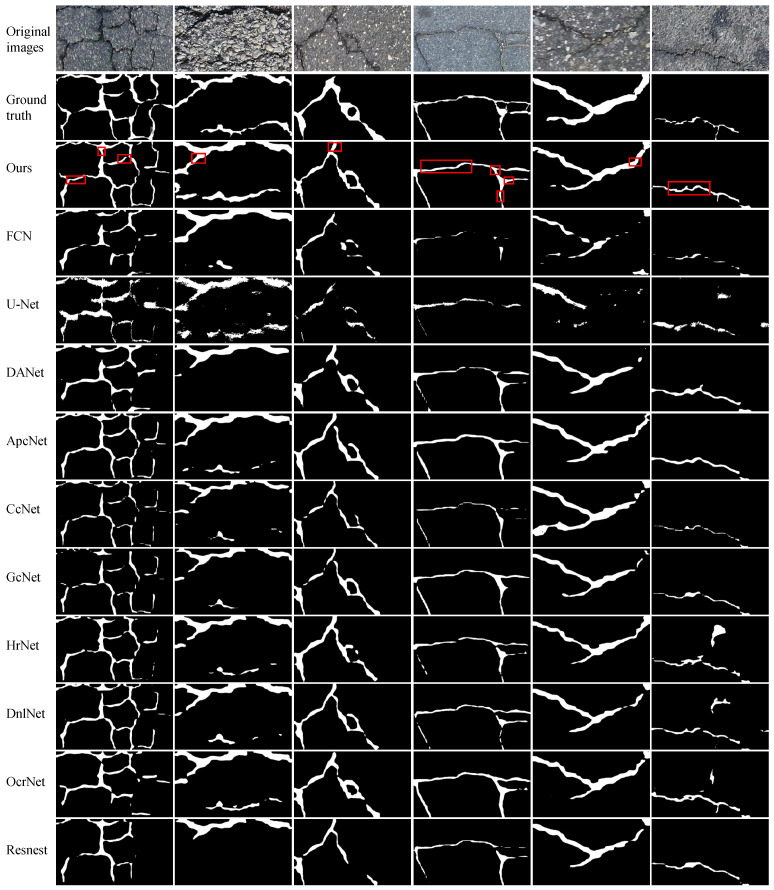
The prediction results obtained by the compared methods used on the Crack500 dataset.

**Figure 12:**
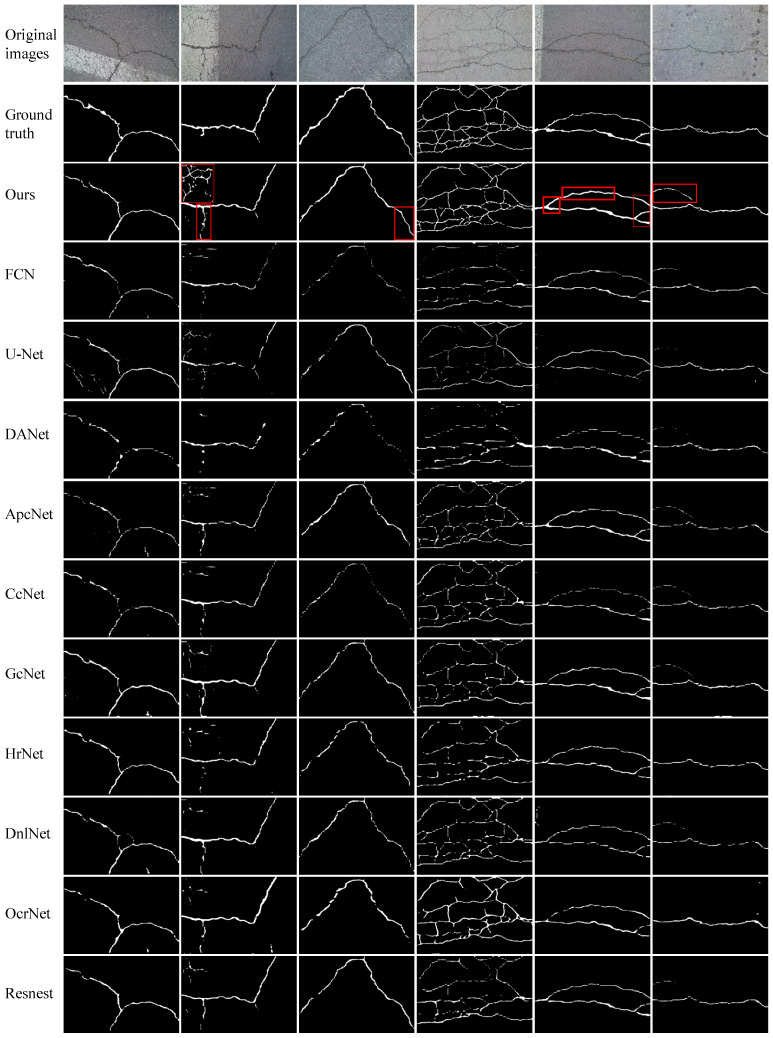
The prediction results obtained by the compared methods used on the CFD dataset.

We perform experiments on four publicly available datasets to evaluate the efficacy of the proposed FetNet method. Detailed comparisons between our FetNet method and 10 crack detection algorithms, including FCN, U-Net, DANet, ApcNet, CcNet, GcNet, HrNet, DnlNet, OcrNet, and Resnest, can be found in Table 1. The evaluation includes the P, R, F1, ACC, mIoU, and frames per second (FPS) metrics obtained for each category. Figure 9, Figure 10, Figure 11 and Figure 12 display the crack segmentation results obtained for typical input images using the proposed method and the comparative methods. The first row contains the original crack images, the second row presents the ground truths, and the subsequent 11 rows showcase the output of the image by the comparative segmentation algorithms. The results showcase the remarkable advantages of the proposed crack segmentation methodology.

Table 1 shows that our proposed FetNet approach significantly outperforms previous segmentation methods, achieving the highest recall of 86.4% and the highest mIoU of 82.7% on the DeepCrack dataset (544×384 pixels). Compared to the second-best method, FetNet achieves more than a 4.2% recall improvement and a 4.1% mIoU improvement. In particular, for less conspicuous cracks in the DeepCrack dataset that are challenging to identify, FetNet still achieves an F1 score of 89.7%, which is more than 3.0% higher than that of the second-best method (ApcNet). From Figure 9, we see that FetNet produces crack segmentation results that closely resemble the labeled image, while the other algorithms produce numerous false segmentation results. This is because the RAM and PFM enhance the model’s ability to capture global information, thereby allowing it to gather more valuable and discriminative crack feature information. The texture unit efficiently retrieves additional meaningful texture and boundary information, resulting in the detection of complete and sharply defined crack outlines. Speed plays a pivotal role in crack segmentation algorithms. Our crack segmentation algorithm achieves a segmentation speed of 60.3 FPS with 544×384 pixel inputs, outperforming the suboptimal HrNet method by 14.1 FPS. In simpler terms, our algorithm takes only 16.5 ms to analyze an image. This indicates that our algorithm strikes an excellent balance between segmentation accuracy and speed.

The crack images in the GAPs384 dataset (1920×1080 pixels) exclusively feature asphalt cracks. As shown in Table 1, FetNet achieves precision, recall, F1 score, mIoU, and FPS results of 83.7%, 71.4%, 73.8%, 69.8%, and 13.2, respectively, outperforming the second-best method by 4.6%, 4.4%, 5.9%, 6.6%, and 5.7 FPS. The ACC of FetNet is slightly lower than that of the best method, the FCN (by 0.3%). As depicted in Figure 10, the images in the GAPs384 dataset exhibit complex topological structures and significant noise interference. Nevertheless, FetNet accurately delineates the contours of cracks, preserving their shapes to the greatest extent possible. In contrast, the FCN, U-Net, and HrNet crack segmentation algorithms are more susceptible to erroneous segmentation results and often misclassify parking lines, pitch, and well lids as crack regions. Due to limited receptive fields, FCN, GcNet, DnlNet, and Resnest tend to assign classifications to specific pixels based only on a few neighboring regions, which can lead to fragmented crack detection and confusion between objects. Consequently, they demonstrate poor detection performance and minimum segmentation quality in the last image. Among these, Resnest yields unsatisfactory segmentation results for all the images, with highly unclear contours. In contrast, FetNet constructs dependency paths based on attention mechanisms to capture long-range global relationships, addressing the limitations of receptive fields.

On the Crack500 dataset (2000×1500 pixels), as shown in Table 1, FetNet achieves precision, recall, F1 score, accuracy, mIoU, and FPS results of 90.4%, 85.3%, 87.9%, 97.1%, 78.6%, and 11.4 FPS, respectively, surpassing the second-best method by 0.9%, 1.0%, 5.0%, 0.2%, 4.6%, and 5.1. Figure 11 illustrates that images present complex crack textures with low contrast levels, and the crack color is similar to the background, making crack features less distinct and prone to false segmentation outcomes. Nevertheless, FetNet effectively integrates global crack features with precise crack boundary information; in this way, it overcomes interference, conforms more closely to crack contours, results in clearer crack segmentation edges, and outperforms the other algorithms. U-Net produces more noise points during segmentation, resulting in fragmented crack representations. This highlights the superior robustness and segmentation ability of FetNet under similar conditions.

On the CFD dataset (480×320 pixels), as shown in Table 1, FetNet achieves precision, recall, F1 score, accuracy, mIoU, and FPS results of 98.8%, 85.3%, 85.7%, 99.2%, 77.6%, and 86.9 FPS, respectively, surpassing the second-best method by 4.5%, 9.3%, 5.1%, 0.2%, 5.8%, and 40.8. As shown in Figure 12, the CFD dataset exhibits relatively minor noise interference, and all methods manage to provide rough segmentation results for the crack contours. Nevertheless, the segmentation results of FetNet are closest to the ground truth, providing a clearer and more accurate segmentation effect. This is because the texture unit plays a crucial role in capturing edge texture information of cracks, leveraging spatial detail information within the feature maps to suppress confusion between cracks and non-cracks, preserving the integrity of the crack contours and rendering them smoother. The other 10 deep learning-based crack segmentation algorithms tend to perform inadequately, especially for detecting edge cracks, sometimes generating false positives.

**Figure 13:**
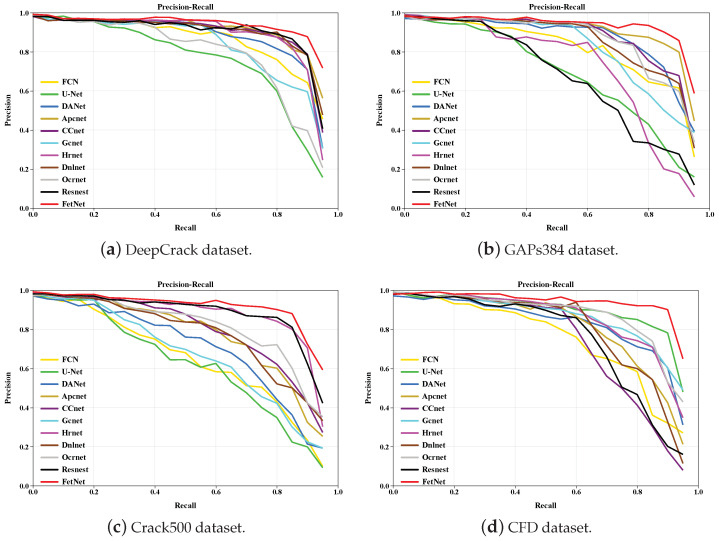
PR curves obtained in the four sets of experiments.

We plot the precision–recall (PR) curves produced by different comparative methods across the DeepCrack, GAPs384, Crack500, and CFD datasets to further demonstrate the performance of the various methods. As shown in Figure 13, FetNet outperforms the other 10 comparative methods, indicating that FetNet has better crack segmentation effectiveness.

### 4.5. Ablation Study

The ablation study in this section was performed on the DeepCrack dataset using Swin-T as the backbone network, and the hyperparameters were consistent across all experiments. Experimental configuration details and quantitative results are recorded in detail in Table 2. To verify the effectiveness of the approach presented in this article, three key modules in the network were evaluated: texture unit, RAM, and PFM. In addition, to verify the effectiveness of LA and ALA, the RAM removal of LA and ALA and the PFM removal of LA and ALA were also considered.

As shown in Table 2, when the texture unit was added, P, mIoU, and F1 scores increased by 2.8%, 3%, and 2.8%, respectively. When RAM was added, P, mIoU, and F1 scores increased by 2.5%, 0.8%, and 1.9%, respectively. When PFM was added, P, mIoU, and F1 scores increased by 2.7%, 1.7%, and 2.6%, respectively. This proves the effectiveness of the texture unit, RAM, and PFM. When LA and ALA were removed from RAM, P, mIoU, and F1 scores decreased by 0.3%, 0.6%, and 0.3%, respectively. When LA and ALA were removed from PFM, P, mIoU, and F1 scores decreased by 0.4%, 0.5%, and 0.4%, respectively. This proves the effectiveness of LA and ALA.

## 5. Conclusions

In this paper, we construct a network for detecting and segmenting road crack images, namely, FetNet. Recognizing the critical importance of contextual and textural information for obtaining accurate segmentation results, we design FetNet based on Swin-T, which captures global context information and incorporates a texture unit and a lightweight convolutional network to simultaneously extract rich textural details and enhance the edge features in high-resolution crack images. A RAM and a PFM are utilized for fusing features of the representations produced by Swin-T and the texture unit, respectively. Furthermore, a CPA is employed to enhance the multiscale receptive fields and fuse the features encountered throughout the framework. Finally, an FC-CRF optimizes the segmentation results, eliminating misclassification and refining the segmentation boundaries. Extensive experiments conducted on four public datasets demonstrate the effectiveness and efficiency of FetNet, which particularly excels on images with complex textures and noise, suggesting that FetNet has significant potential for use in real-time practical applications. In the DeepCrack dataset, FetNet achieves a 4.1% improvement in mIoU compared to the second-best method, ApcNet. For the GAPs384 dataset, FetNet outperforms DANet, the second-best method, by a margin of 6.6% in mIoU. On the Crack500 dataset, FetNet surpasses Resnest, the second-best performer, with a 4.6% increase in mIoU. Finally, in the CFD dataset, FetNet demonstrates a 5.8% improvement in mIoU over the suboptimal method, U-Net. While achieving a relative balance between effectiveness and efficiency, FetNet still has room for improvement. For instance, it is possible to optimize the parameters of Swin-T, optimize model complexity, reduce computing resource requirements, and reduce the dependence on the quality and diversity of training data. This will enable the model to handle more complex scenarios and challenges. Additionally, exploring the applications of FetNet in other domains, such as structural detection and bridge maintenance, is a promising avenue for future research.

## Figures and Tables

**Table 1:** Quantitative results obtained on the DeepCrack, GAPs384, Crack500, and CFD datasets. “**Bold text**” indicates the best result, and “underlined text” indicates the second-best result. ↑ indicates higher is better, and ↓ indicates lower is better.

Methods		FCN [32]	U-Net [33]	DANet [34]	ApcNet [35]	CcNet [36]	GcNet [37]	HrNet [38]	DnlNet [39]	OcrNet [40]	Resnest [41]	Ours
DeepCrack	P↑	93.9	90.4	91.4	85.4	93.9	77.5	89.4	92.8	**95.8**	93.4	95.1
	R↑	76.8	69.7	77.8	82.2	79.9	77.5	81.5	81.5	73.5	80.3	**86.4**
	F1↑	83.1	76.2	83.6	86.7	85.5	82.4	84.9	86.2	80.8	85.6	**89.7**
	ACC↑	97.6	96.3	97.5	97.1	97.8	96.3	97.7	97.8	97.5	97.9	**98.4**
	mIoU↑	74.2	66.9	74.2	78.6	77.1	73.1	76.4	78.0	71.6	77.2	**82.7**
	FPS↑ (ms/img↓)	24.0 (41.7)	22.5 (44.5)	19.2 (52.1)	24.6 (40.7)	24.9 (40.2)	27.3 (36.7)	46.2 (21.7)	24.0 (41.7)	41.1 (24.3)	20.7 (48.3)	**60.3** (**16.5**)
GAPs384	P↑	71.1	69.6	70.4	75.9	76.8	79.1	76.9	78.8	78.3	77.9	**83.7**
	R↑	66.0	60.2	67.0	65.7	63.6	60.5	57.5	62.4	62.8	57.6	**71.4**
	F1↑	65.0	64.7	67.5	69.5	67.9	65.3	61.9	67.3	67.6	61.7	**73.8**
	ACC↑	**98.8**	98.4	98.5	98.1	98.4	98.5	98.1	98.3	98.7	98.4	98.5
	mIoU↑	62.9	58.1	63.2	61.6	60.5	58.6	56.2	60.0	60.3	56.1	**69.8**
	FPS↑ (ms/img↓)	4.3 (232.6)	4.2 (238.1)	3.6 (277.8)	3.7 (270.3)	3.6 (277.8)	4.1 (243.9)	7.5 (133.4)	3.4 (294.2)	6.1 (163.9)	3.0 (333.3)	**13.2** (**75.7**)
Crack500	P↑	73.2	79.1	89.5	87.7	86.5	76.1	83.8	79.4	84.4	85.6	**90.4**
	R↑	70.2	71.9	66.4	76.8	73.2	80.8	81.4	84.3	80.3	80.9	**85.3**
	F1↑	71.6	74.9	72.6	81.2	79.2	78.2	82.6	81.6	82.2	82.9	**87.9**
	ACC↑	94.4	95.3	95.8	96.6	96.5	94.9	96.4	95.8	96.4	96.9	**97.1**
	mIoU↑	62.1	65.3	63.4	71.8	69.7	68.4	73.4	72.1	72.9	74.0	**78.6**
	FPS↑ (ms/img↓)	2.8 (357.2)	2.1 (476.2)	2.2 (454.6)	6.2 (161.3)	4.7 (212.8)	5.3 (188.7)	5.8 (172.4)	6.3 (158.8)	6.1 (163.9)	4.3 (232.6)	**11.4** (**87.7**)
CFD	P↑	82.1	85.3	84.0	76.0	76.5	77.4	78.2	78.7	80.5	74.6	**89.8**
	R↑	69.2	76.8	68.5	76.0	69.3	75.9	71.7	68.4	75.9	70.7	**85.3**
	F1↑	74.0	80.6	73.9	73.5	72.3	76.6	74.5	72.4	78.0	72.5	**85.7**
	ACC↑	98.8	99.0	98.8	98.6	98.6	98.6	98.7	98.6	98.8	98.5	**99.2**
	mIoU↑	65.4	71.8	65.4	65.0	63.9	67.8	65.8	64.0	69.1	64.1	**77.6**
	FPS↑ (ms/img↓)	43.0 (23.3)	29.8 (33.6)	32.4 (30.9)	38.2 (26.2)	27.9 (35.9)	39.5 (25.4)	43.7 (22.9)	41.2 (24.3)	46.1 (21.7)	35.5 (28.2)	**86.9** (**11.5**)

**Table 2:** Results of module ablation study. RAM’ indicates the RAM removal of LA and ALA, and PFM’ indicates the PFM removal of LA and ALA.

Method	Texture Unit	RAM	PFM	RAM’	PFM’	P	mIoU	F1
Swin-T						90.5	75.5	83.5
	✓					93.3	78.5	86.3
		✓				93.0	76.3	85.4
			✓			93.2	77.2	86.1
	✓		✓	✓		94.8	82.1	89.4
	✓	✓			✓	94.7	82.2	89.3
	✓	✓	✓			**95.1**	**82.7**	**89.7**

## Data Availability

The data are unavailable due to privacy security.

## References

[1] Kang D., Benipal S.S., Gopal D.L., Cha Y.J. (2020). Hybrid pixel-level concrete crack segmentation and quantification across complex backgrounds using deep learning. Autom. Constr..

[2] Yang S., Xu Q., Wang Z. (2022). Research progress of structural damage recognition based on convolutional neural networks. J. Archit. Civ. Eng..

[3] Ni T., Zhou R., Gu C., Yang Y. (2020). Measurement of concrete crack feature with android smartphone app based on digital image processing techniques. Measurement.

[4] Choi S., Kim K., Lee J., Park S.H., Lee H.J., Yoon J. (2019). Image processing algorithm for real-time crack inspection in hole expansion test. Int. J. Precis. Eng. Manuf..

[5] Qiao W., Liu Q., Wu X., Ma B., Li G. (2021). Automatic pixel-level pavement crack recognition using a deep feature aggregation segmentation network with a scSE attention mechanism module. Sensors.

[6] Feng D., Zhang Z., Yan K. (2022). A Semantic Segmentation Method for Remote Sensing Images Based on the Swin Transformer Fusion Gabor Filter. IEEE Access.

[7] Peng C., Zhang X., Yu G., Luo G., Sun J. Large kernel matters—Improve semantic segmentation by global convolutional network. Proceedings of the IEEE Conference on Computer Vision and Pattern Recognition.

[8] Vaswani A., Shazeer N., Parmar N., Uszkoreit J., Jones L., Gomez A.N., Kaiser Ł., Polosukhin I. (2017). Attention is all you need. Adv. Neural Inf. Process. Syst..

[9] Liu Z., Lin Y., Cao Y., Hu H., Wei Y., Zhang Z., Lin S., Guo B. Swin transformer: Hierarchical vision transformer using shifted windows. Proceedings of the IEEE Conference on Computer Vision and Pattern Recognition.

[10] Wang L., Li R., Wang D., Duan C., Wang T., Meng X. (2021). Transformer Meets Convolution: A Bilateral Awareness Network for Semantic Segmentation of Very Fine Resolution Urban Scene Images. Remote. Sens..

[11] He K., Zhang X., Ren S., Sun J. Deep residual learning for image recognition. Proceedings of the IEEE Conference on Computer Vision and Pattern Recognition.

[12] Wang X., Hu Z. Grid-based pavement crack analysis using deep learning. Proceedings of the 2017 4th International Conference on Transportation Information and Safety (ICTIS).

[13] Kim B., Yuvaraj N., Sri Preethaa K.R., Arun Pandian R. (2021). Surface crack detection using deep learning with shallow CNN architecture for enhanced computation. Neural Comput. Appl..

[14] Nguyen N.H.T., Perry S., Bone D., Le H.T., Nguyen T.T. (2021). Two-stage convolutional neural network for road crack detection and segmentation. Expert Syst. Appl..

[15] Yang X., Li H., Yu Y., Luo X., Huang T., Yang X. (2018). Automatic pixel-level crack detection and measurement using fully convolutional network. Comput. Civ. Infrastruct. Eng..

[16] Li S., Zhao X., Zhou G. (2019). Automatic pixel-level multiple damage detection of concrete structure using fully convolutional network. Comput. Civ. Infrastruct. Eng..

[17] Hsieh Y.A., Tsai Y.J. (2020). Machine learning for crack detection: Review and model performance comparison. J. Comput. Civ. Eng..

[18] Carion N., Massa F., Synnaeve G., Usunier N., Kirillov A., Zagoruyko S. (2020). End-to-end object detection with transformers. Computer Vision—ECCV 2020, Proceedings of the 16th European Conference, Glasgow, UK, 23–28 August 2020.

[19] Dosovitskiy A., Beyer L., Kolesnikov A., Weissenborn D., Zhai X., Unterthiner T., Dehghani M., Minderer M., Heigold G., Gelly S. (2020). An image is worth 16 × 16 words: Transformers for image recognition at scale. arXiv.

[20] Xie E., Wang W., Yu Z., Anandkumar A., Alvarez J., Luo P. (2021). SegFormer: Simple and efficient design for semantic segmentation with transformers. Adv. Neural Inf. Process. Syst..

[21] Zheng S., Lu J., Zhao H., Zhu X., Luo Z., Wang Y., Fu Y., Feng J., Xiang T., Torr P.H. Rethinking semantic segmentation from a sequence-to-sequence perspective with transformers. Proceedings of the IEEE/CVF Conference on Computer Vision and Pattern Recognition.

[22] Wang W., Su C. (2022). Automatic concrete crack segmentation model based on transformer. Autom. Constr..

[23] Li R., Su J., Duan C., Zheng S. (2020). Linear attention mechanism: An efficient attention for semantic segmentation. arXiv.

[24] Gao J., Geng X., Zhang Y., Wang R., Shao K. (2024). Augmented weighted bidirectional feature pyramid network for marine object detection. Expert Syst. Appl..

[25] Lafferty J., McCallum A., Pereira F. Conditional random fields: Probabilistic models for segmenting and labeling sequence data. Proceedings of the International Conference on Machine Learning.

[26] Cun X., Pun C.M. Image Splicing Localization via Semi-global Network and Fully Connected Conditional Random Fields. Proceedings of the European Conference on Computer Vision (ECCV) Workshops.

[27] Lin T.Y., Goyal P., Girshick R., He K., Dollár P. Focal loss for dense object detection. Proceedings of the IEEE International Conference on Computer Vision.

[28] Liu Y., Yao J., Lu X., Xie R., Li L. (2019). DeepCrack: A deep hierarchical feature learning architecture for crack segmentation. Neurocomputing.

[29] Eisenbach M., Stricker R., Seichter D., Amende K., Debes K., Sesselmann M., Ebersbach D., Stoeckert U., Gross H.M. How to get pavement distress detection ready for deep learning? A systematic approach. Proceedings of the 2017 International Joint Conference on Neural Networks (IJCNN).

[30] Yang F., Zhang L., Yu S., Prokhorov D., Mei X., Ling H. (2019). Feature pyramid and hierarchical boosting network for pavement crack detection. IEEE Trans. Intell. Transp. Syst..

[31] Shi Y., Cui L., Qi Z., Meng F., Chen Z. (2016). Automatic road crack detection using random structured forests. IEEE Trans. Intell. Transp. Syst..

[32] Shelhamer E., Long J., Darrell T. (2017). Fully convolutional networks for semantic segmentation. IEEE Trans. On Pattern Anal. Mach. Intell..

[33] Ronneberger O., Fischer P., Brox T. (2015). U-net: Convolutional networks for biomedical image segmentation. Medical Image Computing and Computer-Assisted Intervention–MICCAI 2015, Proceedings of the 18th International Conference, Munich, Germany, 5–9 October 2015.

[34] Fu J., Liu J., Tian H., Li Y., Bao Y., Fang Z., Lu H. Dual attention network for scene segmentation. Proceedings of the IEEE/CVF Conference on Computer Vision and Pattern Recognition.

[35] He J., Deng Z., Zhou L., Wang Y., Qiao Y. Adaptive pyramid context network for semantic segmentation. Proceedings of the IEEE/CVF Conference on Computer Vision and Pattern Recognition.

[36] Huang Z., Wang X., Huang L., Huang C., Wei Y., Liu W. Ccnet: Criss-cross attention for semantic segmentation. Proceedings of the IEEE/CVF International Conference on Computer Vision.

[37] Cao Y., Xu J., Lin S., Wei F., Hu H. Gcnet: Non-local networks meet squeeze-excitation networks and beyond. Proceedings of the IEEE/CVF International Conference on Computer Vision Workshops.

[38] Sun K., Xiao B., Liu D., Wang J. Deep high-resolution representation learning for human pose estimation. Proceedings of the IEEE/CVF Conference on Computer Vision and Pattern Recognition.

[39] Yin M., Yao Z., Cao Y., Li X., Zhang Z., Lin S., Hu H. Disentangled non-local neural networks. Proceedings of the Computer Vision–ECCV 2020: 16th European Conference.

[40] Yuan Y., Chen X., Wang J. Object-contextual representations for semantic segmentation. Proceedings of the Computer Vision–ECCV 2020: 16th European Conference.

[41] Zhang H., Wu C., Zhang Z., Zhu Y., Lin H., Zhang Z., Sun Y., He T., Mueller J., Manmatha R. Resnest: Split-attention networks. Proceedings of the IEEE/CVF Conference on Computer Vision and Pattern Recognition.

